# Higher 3-year recurrence-free survival rate in patients with complete pathological remission following neoadjuvant chemotherapy plus immunotherapy for esophageal cancer: a two-center, propensity score matching study

**DOI:** 10.3389/fonc.2024.1463936

**Published:** 2025-01-14

**Authors:** Hai Zhang, Haiquan He, Qingyi Feng, Bomeng Wu, Ying Chen, Zhenyang Zhang, Linrong Zhou, Cui Li, Wanli Lin, Jiangbo Lin

**Affiliations:** ^1^ Department of Thoracic Surgery, Fujian Medical University Union Hospital, Fuzhou, China; ^2^ The Graduate School of Fujian Medical University, Fuzhou, China; ^3^ Department of Thoracic Surgery, Gaozhou People’s Hospital, Guangdong Esophageal Cancer Institute Gaozhou, Gaozhou, China

**Keywords:** esophageal squamous cell carcinoma, immunotherapy, neoadjuvant chemoradiotherapy, pathological complete response, recurrence-free survival

## Abstract

**Background:**

Neoadjuvant therapy is preferentially recommended for resectable locally advanced esophageal malignancies, with patients who achieve pathological complete response (PCR) anticipated to have longer survival rates. The aim of this study was to compare 3-year follow-up data for patients with esophageal malignancy who achieved PCR through neoadjuvant chemotherapy (nCRT) and to compare the findings with those of neoadjuvant immunotherapy plus chemotherapy (nICT).

**Methods:**

This retrospective study included 85 patients with esophageal cancer who underwent surgical resection following nCRT (n=47) or nICT (n=38) between January 1, 2016 and January 1, 2020 at Fujian Medical University Union Hospital and Gaozhou People’s Hospital. Propensity score matching was used to match baseline data and reduce bias between the patient groups. Data during the neoadjuvant treatment and perioperative periods were compared, and follow-up was performed to evaluate differences in 3-year survival rate and recurrence-free survival.

**Results:**

After propensity score matching, 28 nCRT patients and 38 nICT patients were included. During neoadjuvant therapy, the nCRT group had higher incidences of leukopenia and neutropenia than did the nICT group. No significant differences were observed in the incidences of hemoglobin decrease, platelet decrease, liver function damage, elevated serum creatinine, diarrhea, radioactive pneumonia or immunotherapy-related pneumonia, and esophageal perforation. The nCRT group had fewer lymph node dissections and lymph node stations. Postoperative lung infection (50.00%) was significantly higher in the nICT group than in the nCRT group (25.00%). The 3-year survival rates were 97.37% and 85.71% in the nICT and nCRT groups, respectively; the 3-year recurrence-free survival rate was significantly lower in the nCRT group (82.14%) than in the nICT group (97.37%, P=0.02).

**Conclusions:**

These findings suggest that patients with esophageal cancer who achieve PCR after nICT treatment may have lower rates of disease recurrence.

## 1 Introduction

Currently, neoadjuvant therapy is preferentially recommended for patients with surgically resectable locally advanced esophageal malignancies (cT4N0M0, CT1-3N+M0) to enhance R0 resection rates and improve patient survival (1). Clinical guidelines commonly recommend two neoadjuvant therapy modalities: neoadjuvant chemoradiotherapy (nCRT) and neoadjuvant chemotherapy (nCT) (2, 3). Pathological complete response (PCR) rates for nCT and nCRT have been reported as 3%–32% and 30%–50%, respectively (4). PCR rate is an indicator commonly used to evaluate the effectiveness of neoadjuvant therapy and may indicate favorable event-free survival (EFS) and disease-free survival (DFS) outcomes (5). However, the correlation between a higher PCR rate and longer survival, as well as the use of PCR as an alternative indicator to assess the efficacy of new adjuvant therapies, remains controversial (6). Therefore, determining the optimal approach to neoadjuvant therapy has become an important topic in clinical discussions, prompting extensive research in clinical practice. In 2020, it was initially reported that immune checkpoint inhibitors could improve long-term overall survival (OS) outcomes in advanced esophageal cancer (7). Subsequent studies have confirmed their safety and effectiveness in this context (8, 9). Regarding postoperative adjuvant therapy direction, immune checkpoint inhibitors have been associated with reduced risk of disease recurrence and death when compared with a placebo (10). This finding has led to increasing phase II or III clinical trials focusing on combination approaches involving neoadjuvant immunotherapy plus chemotherapy (nICT) for locally advanced resectable esophageal malignancies (11, 12). In contrast to nCRT, which primarily focuses on directly eliminating tumor cells and modifying the microenvironment, nICT emphasizes the activation of the host’s immune system to target tumors through immune cells. The PCR rate is a commonly evaluated parameter to assess the effectiveness of neoadjuvant therapy for malignant tumors in clinical practice. It has also been correlated with DFS and OS rates of patients (13). Compared with surgery alone, neoadjuvant therapy combined with surgery for the treatment of locally advanced esophageal squamous cell carcinoma can enhance the complete surgical resection rate of the tumor and improve the long-term survival rate of patients (14). However, relevant studies conducted to investigate the potential disparities in achieving PCR among various neoadjuvant therapy modalities, compare neoadjuvant therapy cycles with the perioperative period, and assess tumor recurrence and long-term survival are lacking.

The aim of the present study was to compare nCRT alone with the currently popular combination, nICT, in patients with locally advanced resectable esophageal cancer who achieved PCR. Our goal was to explore the efficacy of nICT for the treatment of locally advanced esophageal malignancies.

## 2 Materials and methods

### 2.1 Ethics approval

This study was approved by the Institutional Review Board of Union Hospital affiliated with Fujian Medical University (approval number: 2023KY241) and the Ethical Committee of Gaozhou People’s Hospital (approval number: GYLLPJ-2022104). The need for written informed consent was waived because of the retrospective nature of the study.

### 2.2 Study participants

**Figure 1:**
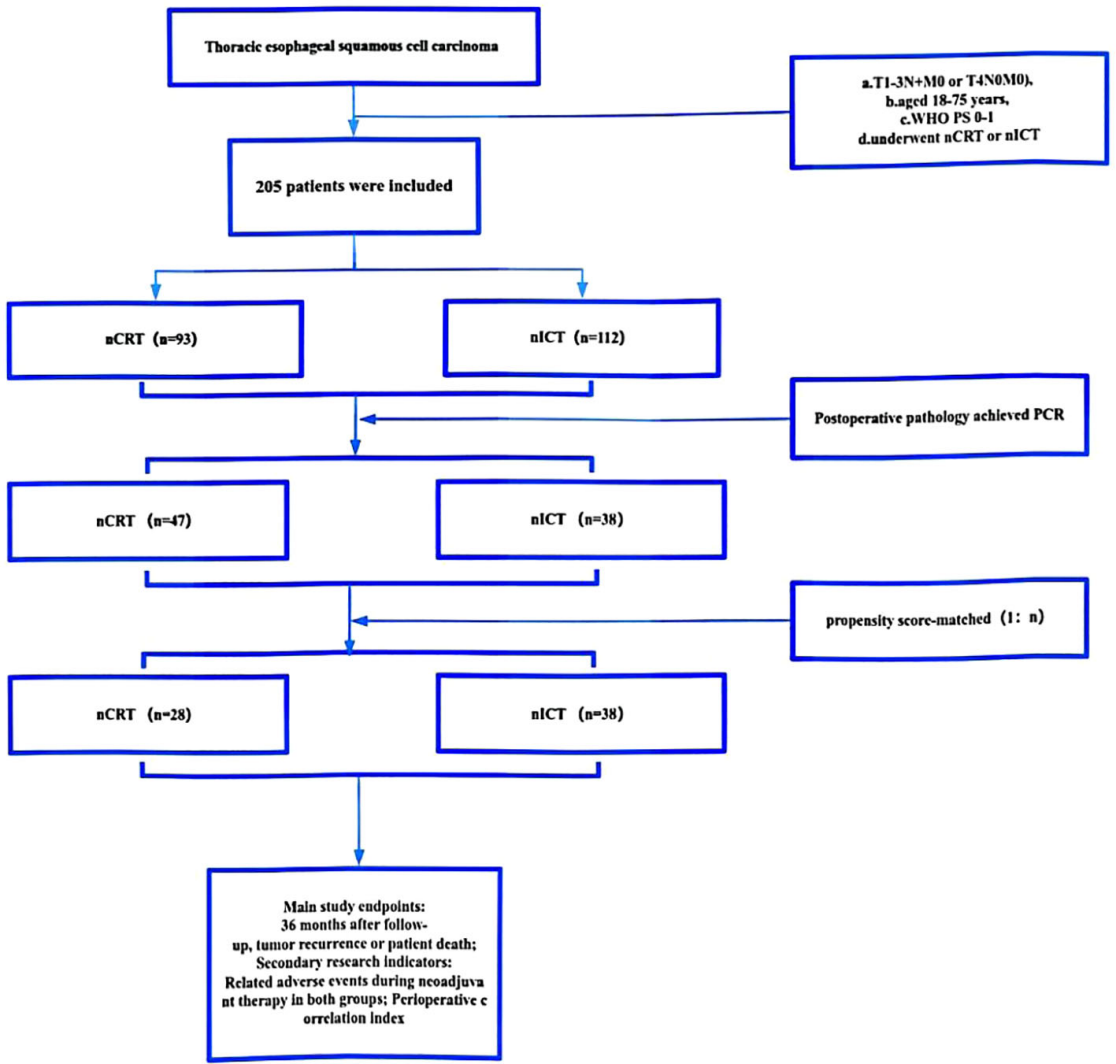
Study flowchart. The study included a total of 205 patients aged between 18 and 75 years who were diagnosed with resectable esophageal squamous cell carcinoma and received neoadjuvant therapy with either cT4N0M0 or cT1-3N+M0. The patients were divided into two groups based on the type of neoadjuvant therapy: nCRT group (n=93) and nICT group (n=112). In total, 47 patients in the nCRT group and 38 in the nICT group achieved PCR. After applying the 1:n propensity score matching method, 28 and 38 patients were included in the nCRT and nICT groups, respectively. A comparative analysis was conducted to evaluate the neoadjuvant period, perioperative period, and 3-year follow-up outcomes between the two groups. nCRT, neoadjuvant chemoradiotherapy; nICT, neoadjuvant immunotherapy plus chemotherapy; WHO, World Health Organization; PS, performance status; PCR, pathological complete response.

This study included patients with malignant esophageal tumors who underwent neoadjuvant therapy and surgical resection at Fujian Medical University Union Hospital and Gaozhou People’s Hospital between January 1, 2016 and January 1, 2020. The inclusion criteria were as follows: 1) age 18–75 years; 2) confirmed diagnosis of esophageal squamous cell carcinoma using electronic gastroscopy biopsy pathology; 3) clinical staging of either cT4N0M0 or cT1-3N+M0 esophageal malignant tumors, confirmed as resectable by enhanced cervical and upper abdominal computed tomography (CT) scan, ultrasonic gastroscopy, or PET-CT; 4) preoperative nCRT or nICT; 5) surgical treatment using the McKeown procedure; 6) postsurgical data indicating PCR; and 7) availability of complete follow-up data. **Figure 1** illustrates the research process.

### 2.3 Neoadjuvant therapy and operative program

NCRT regimen: This regimen included radiotherapy using an appropriate intensity-modulated radiotherapy technique. The regimen involved 20–24 sessions with a dose of 40–54 Gy/20–24 F for the target volume (GTV) and 40–44 Gy/20 F for the clinical target volume (CTV). Additionally, patients received paclitaxel chemotherapy drugs (paclitaxel injection 135–175 mg/m^2^, docetaxel injection 75 mg/m^2^, or paclitaxel albumin-bound formulation 260 mg/m^2^), fluorouracil (750–1000 mg/m^2^ d1–d4), or gemcitabine (25 mg/m^2^ d1, 8, 15, 22) in combination with platinum-based chemotherapy drugs (cisplatin 60–100 mg/m^2^ or carboplatin 0.3–0.4 g/m^2^ or nedaplatin 80–100 mg/m^2^). Chemotherapy drugs are used for 2–4 cycles, with dose adjustments made based on specific drugs and patient responses. Surgery was performed 6–8 weeks after the completion of nCRT.

NICT regimen: The chemotherapy regimen (q3w) was identical to that used for neoadjuvant concurrent chemoradiotherapy. Immunotherapeutic drugs (nivolumab 3 mg/kg, d1, q3w; pembrolizumab 2 mg/kg, d1, q3w; camrelizumab 200 mg, d1, q3w; sintilimab 200 mg, d1, q3w; and toripalimab 200 mg, d1, q3w) were administered prior to chemotherapy.

The surgical treatment plan involved the adoption of a minimally invasive or open McKeown approach. Routine dissection of two fields was performed to clear the mediastinal and upper abdominal lymph nodes. For patients with suspected cervical lymph node metastasis based on preoperative evaluation, three-field dissection was chosen, which included clearance of the cervical, mediastinal, and upper abdominal drainage lymph nodes.

### 2.4 Observation indexes

The general demographic and clinical characteristics of the patients prior to treatment, including age, sex, location of the esophageal tumor, and clinical stage according to the eighth edition of the AJCC/NCCN guidelines, were recorded.

The occurrence of adverse events, such as gastrointestinal reactions, hematological toxicities, liver and kidney function impairment, radiation pneumonia, immune-related pneumonia, and esophageal fistulas, during the neoadjuvant therapy cycle were documented. Adverse events were graded using the CTCAE version 5.0 classification method (http://ctep.cancer.gov).

Following neoadjuvant treatment, clinical efficacy and post-treatment stage were evaluated using neck and chest plus upper abdomen CT enhancement, PET-CT, or ultrasonic gastroscopy 1 week before surgery. Efficacy evaluation was based on RECIST1.1 criteria for solid tumors.

Perioperative clinical data included operation time, intraoperative blood loss, postoperative pathological lymph node retraction rate, postoperative drainage time and thoracic drainage volume, postoperative intensive care unit (ICU) length of stay and rate of return to ICU, postoperative length of hospital stay, incidence of surgery-related complications, and 30-day postoperative mortality. After a 3-year follow-up period, 3-year survival rates and postoperative tumor recurrence rates were calculated for both groups.

### 2.5 Statistical analysis

Statistical analysis was performed using SPSS 23.0 (IBM Corporation, Armonk, NY, USA). Mean ± standard deviation was calculated for the following variables: age, operative time, intraoperative blood loss, number of lymph node dissections, number of lymph node dissection stations, chest tube drainage time, thoracic drainage volume, length of ICU stay, and postoperative length of hospital stay. Student’s t-test was used to analyze these variables. Pearson’s chi-squared test was used to evaluate the following variables: gender, tumor location, rate of neoadjuvant therapy-induced PCR, lymph node response, and pulmonary infection. The Mann–Whitney U test was used to assess tumor grade, cTNM stages, hemoglobin decrease, leukopenia, neutropenia, thrombocytopenia, total bilirubin increase, glutamic pyruvic transaminase increase, glutamic oxaloacetic transaminase increase, and elevated serum creatinine. Fisher’s exact test was used to analyze diarrhea, pneumonia (radiation pneumonia or immune-associated pneumonia), esophageal perforation, ICU return rate, respiratory failure, heart failure, arrhythmia, thoracogastric fistula, bronchial fistula, anastomotic fistula, stroke, trachyphonia, death (30 days after surgery) and tumor recurrence. The likelihood ratio test was used to compare death rates between the two groups. Kaplan–Meier survival curves were generated using GraphPad Prism 9 (GraphPad Software, La Jolla, CA, USA) to analyze 3-year recurrence-free survival rates. Results were deemed statistically significant when the p-value was <0.05.

## 3 Results

This study involved a total of 205 patients from two centers. Among these patients, 93 received nCRT, and 47 (50.54%) of them achieved PCR. Additionally, 112 patients received nICT, and 38 (33.93%) of them achieved PCR. A comparison between the two groups revealed a chi-square value of 5.77 and a p-value of 0.01.

A 1:n propensity score matching method was employed for the two patient groups. Covariates included age, sex, tumor location, and clinical stage of the tumor. In the analysis, the treatment and control groups were paired using 1:.35 nearest neighbor matching method to ensure that the two groups were balanced in terms of the covariates. Eventually, 66 patients were included in the study: 28 received nCRT and 38 received nICT (**Figure 1**). Comparison of the demographic characteristics and clinical stages before and after propensity score matching is shown in **Table 1**.

**Table 1:** Comparison of demographic characteristics and clinical stage before and after propensity scoring.

Variables	Before matching		*χ^2^*/*t*/Z	P	After matching		*χ^2^*/*t*/Z	P
	nRCT=47(%)	nICT=38(%)			nRCT=28(%)	nICT=38(%)		
Age	61.09 ± 8.12	62.03 ± 6.42	-0.58	0.56	61.11 ± 8.85	62.03 ± 6.42	-0.46	0.64
Gender(%)			0.31	0.63			0.26	0.60
male	32(68.09)	28(73.68)			19(67.86)	28(73.68)		
female	15(31.91)	10(26.32)			9(32.14)	10(26.32)		
Tumor location(%)			6.25	0.04			2.40	0.30
up	10(21.28)	5(13.16)			3(10.71)	5(13.16)		
middle	33(70.21)	22(57.89)			21(75.00)	22(57.89)		
Low	4(8.51)	11(28.95)			4(14.29)	11(28.95)		
Clinical stage of tumor(%)								
G			-1.30	0.19			-0.45	0.70
G1	14(29.79)	16(42.11)			11(39.29)	16(42.11)		
G2	20(42.55)	15(39.47)			10(35.71)	15(39.47)		
G3	13(27.66)	7(18.42)			7(25.00)	7(18.42)		
T			-0.02	0.97			-0.40	0.77
T2	10(21.28)	7(18.42)			5(17.86)	7(18.42)		
T3	34(72.34)	30(78.95)			21(75.00)	30(78.95)		
T4	3(6.38)	1(2.63)			2(7.14)	1(2.63)		
N			-0.26	0.79			-0.74	0.49
N0	5(10.64)	8(21.05)			2(7.14)	8(21.05)		
N1	34(72.34)	21(55.26)			20(71.42)	21(55.26)		
N2	6(12.77)	7(18.42)			4(14.29)	7(18.42)		
N3	2(4.25)	2(5.27)			2(7.14)	2(5.27)		
Clinical stages(%)			-1.27	0.20			-1.83	0.07
II	10(21.28)	13(34.21)			4(14.29)	13(34.21)		
III	32(68.08)	22(57.89)			20(71.42)	22(57.89)		
IVa	5(10.64)	3(7.90)			4(14.29)	3(7.90)		

Data are presented as n (%) or mean ± SD. nCRT, neoadjuvant hemoradiotherapy; nICT, neoadjuvant immunotherapy plus chemotherapy; G, Grade; T, Tumor; N, Node; M, Metastasis.

During the neoadjuvant treatment period, the nCRT group experienced a higher incidence of hematological toxicity, specifically decreases in white blood cell and neutrophil counts, with statistically significant differences between the groups (p<0.05). However, the differences in the occurrence rates of reduced hemoglobin and platelet levels were not statistically significant (p>0.05). Liver function damage was evaluated using biochemical indicators, including aspartate transaminase, alanine transaminase, and total bilirubin levels. Damage to kidney function was assessed based on serum creatinine levels. These biochemical indicators did not differ significantly between the two groups (p>0.05). In terms of imaging-related indicators, two cases of radiation pneumonia occurred in the nCRT group, whereas no cases of immunotherapy-related pneumonia were observed during the neoadjuvant treatment period. Additionally, one case of esophageal fistula occurred in the nCRT group during neoadjuvant therapy. The difference in the incidence of diarrhea between the two groups was not statistically significant (**Table 2**).

**Table 2:** Comparison of relevant indicators during the neoadjuvant treatment.

Variables	nCRT (n=28)	nICT (n=38)	Z	p
Hemoglobin decreased(%)			-1.07	0.29
Grade0	17(60.71)	27(71.05)		
Grade1	6(21.43)	9(23.68)		
Grade2	5(17.86)	1(2.63)		
Grade3	0(0.00)	1(2.63)		
Leukopenia(%)			-4.06	0.001
Grade0	7(25.00)	26(68.42)		
Grade1	6(21.43)	7(18.42)		
Grade2	6(21.43)	5(13.16)		
Grade3	8(28.57)	0(0.00)		
Grade4	1(3.57)	0(0.00)		
Neutropenia(%)			-3.28	0.001
Grade0	13(46.43)	32(84.21)		
Grade1	8(28.57)	4(10.53)		
Grade2	3(10.71)	1(2.63)		
Grade3	3(10.71)	1(2.63)		
Grade4	1(3.57)	0(0.00)		
Thrombocytopenia(%)			-1.69	0.15
Grade0	24(85.71)	37(97.37)		
Grade1	4(14.29)	0(0.00)		
Grade2	0(0.00)	1(2.63)		
Total bilirubin increased(%)			-1.22	0.50
Grade0	28(100.00)	36(94.74)		
Grade1	0(0.00)	2(5.26)		
Glutamic pyruvic transaminase increased(%)			-0.05	0.96
Grade0	21(75.00)	28(73.68)		
Grade1	4(14.29)	8(21.05)		
Grade2	1(3.57)	2(5.26)		
Grade3	2(7.14)	0(0.00)		
Glutamic oxaloacetic transaminase increased(%)			-0.69	0.53
Grade0	23(82.14)	28(73.68)		
Grade1	3(10.71)	8(21.05)		
Grade2	1(3.57)	2(5.26)		
Grade3	1(3.57)	0(0.00)		
Elevated serum creatinine(%)			-0.31	0.75
Grade0	26(92.86)	36(74.74)		
Grade1	2(7.14)	2(5.26)		
Diarrhea(%)	1(3.57)	0(0.00)	/	0.42
Pneumonia(Radiation pneumonia or immune-associated pneumonia)(%)	1(3.57)	0(0.00)	/	0.42
Esophageal perforation(%)	1(3.57)	0(0.00)	/	0.42

Both groups underwent the combined thoracoscopic and laparoscopic McKeown procedure. The nICT group had higher numbers of lymph nodes and lymph node station dissections. Postoperative pulmonary infections occurred in both groups, with the nICT group having the highest incidence (50%). Several patients in both groups died within 30 days after surgery. In the nCRT group, one death occurred due to a thoracic gastric fistula complicated by severe pneumonia, and another occurred due to severe postoperative immune-related pneumonia. Perioperative treatment indicators and postoperative complication rates in both groups are shown in **Table 3**.

**Table 3:** Comparison of perioperative indicators.

Variables	nCRT group (n=28)	nICT group (n=38)	t/x2	p
Intraoperative indicators				
Operation time(min)	342.71 ± 77.93	313.29 ± 63.62	1.68	0.09
Intraoperative blood loss(ml)	86.43 ± 44.32	85.26 ± 46.72	0.10	0.91
Number of dissected lymph nodes	26.50 ± 12.49	35.95 ± 16.06	-2.58	0.01
Number of dissected lymph nodes stations	10.32 ± 3.67	12.18 ± 2.54	-2.31	0.02
Postoperative related indicators				
Lymph node response(%)	5(17.86)	12(31.58)	1.59	0.20
Chest tube drainage time (d)	6.75 ± 3.87	9.05 ± 6.18	-1.736	0.08
Chest tube drainage volume (ml)	1314.50 ± 1106.87	1603.89 ± 1135.66	-1.03	0.30
ICU stay (h)	8.16 ± 10.65	13.82 ± 43.64	-0.67	0.50
Return to the ICU (%)	1(3.57)	2(5.26)	/	1.00
Postoperative hospital stay (d)	11.32 ± 4.79	12.76 ± 8.40	-0.81	0.41
Postoperative complications				
Respiratory failure(%)	1(3.57)	1(2.63)	/	1.00
Heart failure(%)	1(3.57)	0(0.00)	/	0.42
Arrhythmia (%)	1(3.57)	4(10.53)	0.34	0.55
Thoracogastric fistula (%)	1(3.57)	1(2.63)	/	1.00
Bronchial fistula (%)	1(3.57)	0(0.00)	/	0.42
Anastomotic fistula (%)	2(7.14)	3(7.89)	/	1.00
Pulmonary infection(%)	7(25.00)	19(50.00)	4.22	0.04
Stroke(%)	1(3.57)	0(0.00)	/	1.000
Trachyphonia (%)	1(3.57)	0(0.00)	/	1.000
Death (30 days after surgery)(%)	1(3.57)	1(2.63)	/	1.000

ICU, Intensive Care Unit.

**Figure 2:**
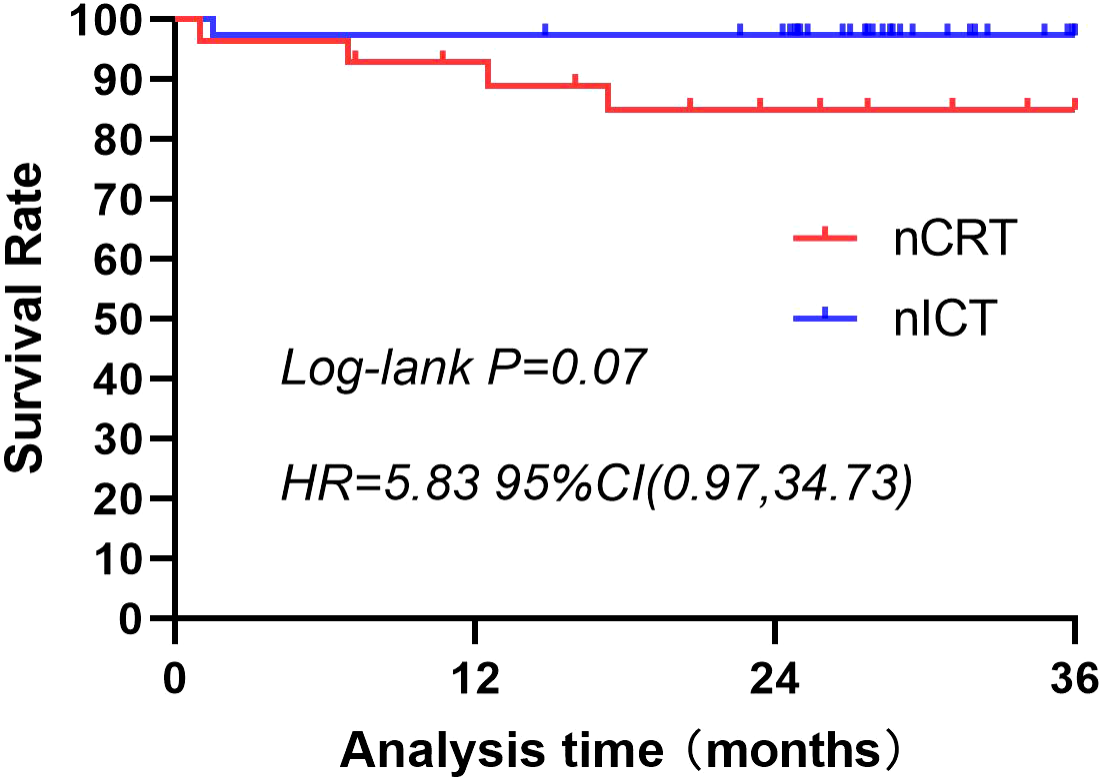
Three-year survival rates for patients treated with nCRT or nICT before surgical resection of esophageal squamous cell carcinoma. The 3-year survival rate is higher in the nICT group (97.37%) than in the nCRT group (85.71%); however, the difference is not statistically significant (p=0.07; HR=5.83; 95%CI: [0.97, 34.73]). nCRT, neoadjuvant chemoradiotherapy; nICT, neoadjuvant immunotherapy plus chemotherapy; HR, hazard ratio; CI, confidence interval.

**Figure 3:**
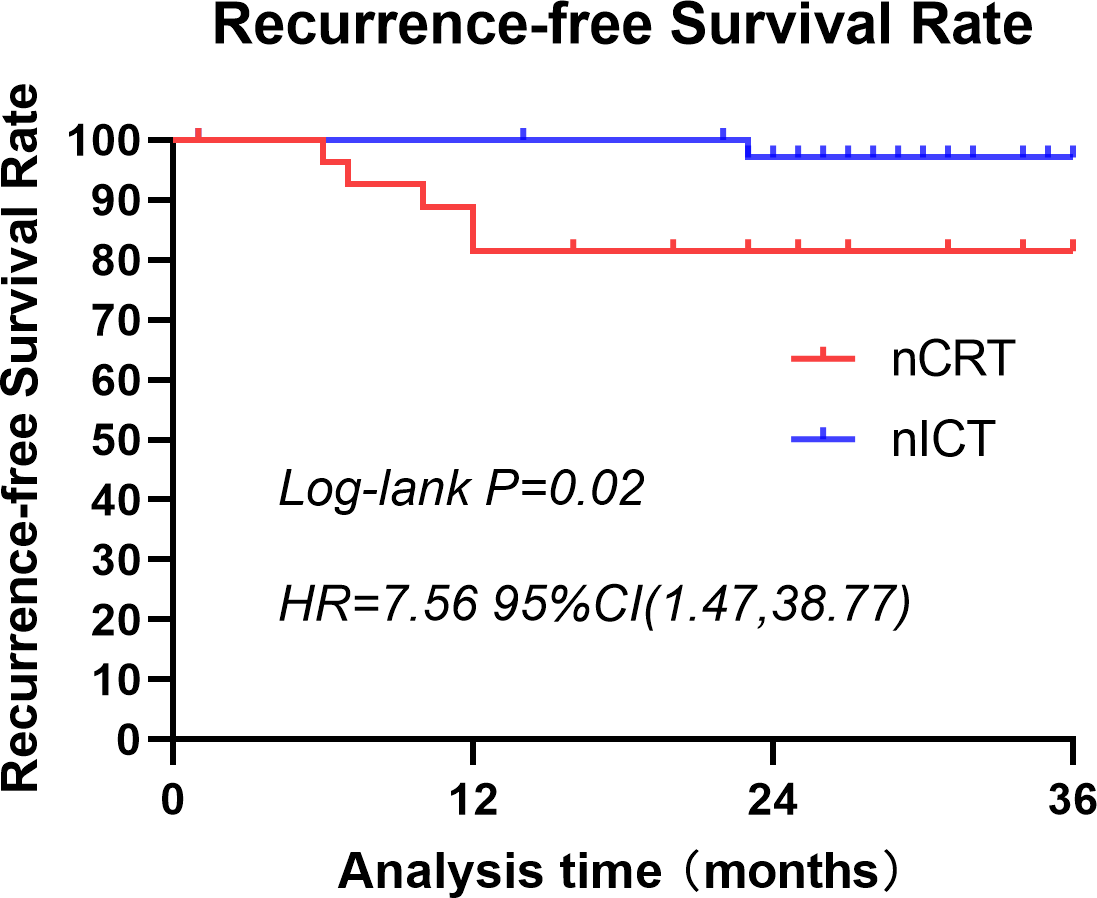
Three-year recurrence-free survival rates for patients treated with nCRT or nICT before surgical resection of esophageal squamous cell carcinoma. This graph illustrates the recurrence-free survival of patients in the nCRT and nICT groups over a span of 3 years. There is a statistically significant difference between the two groups (p=0.02; HR=7.56; 95% CI: [1.47, 38.77]). nCRT, neoadjuvant chemoradiotherapy; nICT, neoadjuvant immunotherapy plus chemotherapy; HR, hazard ratio; CI, confidence interval.

A 3-year follow-up was conducted for both patient groups. In the nCRT group, four deaths occurred within 3 years, compared with only one in the nICT group. Three-year survival rate was higher in the nICT group (97.37% vs. 85.71%); however, the difference was not statistically significant (**Figure 2**). Within these 3 years, the nCRT group recorded five cases of recurrence, including three instances of distant metastasis. In contrast, the nICT group had only one case of anastomotic site recurrence, and the difference between the two groups was statistically significant (P=0.02). Comparison of recurrence-free survival rates between the two groups is illustrated in **Figure 3**.

## 4 Discussion

The PCR rate for esophageal squamous cell carcinoma following nCRT was reported to be 49% in the CROSS study (15) and 43% in the NEOCRTEC5010 study (14). In the present study, a PCR rate of 50.54% was observed in the nCRT group. Current evidence indicates that nCRT for the treatment of locally advanced esophageal squamous cell carcinoma can yield a higher rate of pathological remission than nICT alone (4, 16). However, real-world data have shown that the acceptance rate of nCRT in the clinical space is low, particularly in elderly patients (17). This may be attributable to three reasons. First, these data indicate that, although nCRT yields better PCR results, it does not confer long-term survival benefits, compared with nCT alone (18). Second, the delineation of the radiotherapy target area for nCRT remains controversial. Clinically, two delineation modes are commonly adopted: elective node irradiation (ENI), as shown in the CROSS study (15), and subclinical lesion irradiation (SLI), as defined in the NEOCRTEC5010 study (14). These different approaches may lead to variations in the irradiation level of at-risk organs, such as the lungs, heart, and spinal cord. Nevertheless, both radiotherapy plans were effective. Finally, there was a high incidence of adverse events during neoadjuvant therapy and in the perioperative period. In this study, leukopenia and neutropenia were the principal adverse events observed in the nCRT group. Among the patients who underwent surgery after nCRT, the most common hematological toxicities were leukopenia (6%) and neutropenia (2%) (15). This may be because radiotherapy itself inhibits the hematopoietic function of the bone marrow, resulting in reduced production of white blood cells and neutrophils and, consequently, hematological toxicity. In contrast, nICT may also have a certain impact on the immune system; however, because the mechanism of immunotherapy is different from that of radiotherapy, its inhibition of bone marrow hematopoietic function may be relatively weak, resulting in a relatively low incidence of hematological toxicity. Patients seeking medical treatment for esophageal squamous cell carcinoma are often elderly, exhibit high nutritional and physiological status assessment scores, and undergo a prolonged cycle of nCRT. During this therapy, patients’ physiological status may be further compromised due to their limited medical experience. Additionally, a longer duration of neoadjuvant therapy increases the likelihood of complications arising during treatment. Consequently, patients are burdened with elevated medical expenses and time commitments, leading to decreased compliance. Donlon et al. (19) demonstrated that patients’ adoption of the CROSS research scheme led to an increase in the incidence of muscle atrophy from 14% to 30%. In their study, 8% of patients did not undergo the planned surgery, and 13% of the patients who underwent surgery experienced postoperative respiratory failure. In our study, two patients developed radiation pneumonitis during neoadjuvant treatment. Regarding the rate of postoperative complications, we observed two cases of perioperative death in the nCRT group, consistent with the results of van Hagen et al. (15), which indicated a 4% postoperative mortality rate.

nICT is an emerging neoadjuvant therapy that has gained traction in the past 5 years, and numerous clinical centers are actively investigating its safety, feasibility, and efficacy. The NCT04006262 study (20) showed that, for patients with dMMR/MSI-H gastric or esophagogastric junction tumors, neoadjuvant immunotherapy could achieve high PCR rates without increasing treatment-related toxicity. A meta-analysis of 27 clinical studies revealed an overall PCR rate of 32.4% (95% confidence interval [CI], 28.2%–36.8%) in patients with esophageal squamous cell carcinoma (21). Notably, the neoadjuvant immunotherapy group did not show a significant increase in blood toxicity, immune-associated pneumonia, or immune-associated rash during treatment. Additionally, the perioperative time was shorter than that of nCRT, suggesting that the surgical procedures may be less challenging than those associated with nCRT. Our study showed that surgical lymph node dissection after nCRT typically resulted in fewer nodes and fewer stations of dissection than did surgical lymph node dissection after nCRT, mainly because the local effect of radiotherapy may have resulted in shrinkage of the lymph nodes or changes in their structure, making it difficult to identify and remove more lymph nodes during surgery. Immunotherapy works by activating or enhancing the body’s own immune system, and this enhanced immune response may reduce the degree of lymph node involvement. Together, these factors may have contributed to the difference in the number of lymph nodes and the number of stations removed during surgery. In patients who achieved PCR after receiving nICT, a slightly higher incidence of perioperative pulmonary infection was observed. This may be related to the inhibitory effect of chemotherapy drugs on the immune system and the changes in immune response caused by immunotherapy. Further research is required to confirm the specific cause and clinical significance of this phenomenon. However, no increase in perioperative mortality was observed, despite the nICT group having a lower PCR rate than the nCRT group. Ongoing clinical exploration of this approach persists due to its low toxicity profile and high efficiency. Based on these findings, which highlight the favorable balance between low toxicity and high efficacy associated with this approach, clinical investigations into this mode continue to advance.

nCRT has yielded a favorable survival rate in patients with esophageal squamous cell carcinoma, compared with surgery alone. A study conducted at eight clinical research centers in the Netherlands (22) revealed that patients with esophageal squamous cell carcinoma who underwent nCRT with surgery had a median overall survival time of 86.1 months, whereas those who underwent surgery alone had a median overall survival time of 21.1 months (hazard ratio [HR] 0.48 [95%CI 0.28–0.83]; log-rank p = 0.008). Long-term follow-up studies have confirmed that nCRT provides an OS benefit in patients with resectable esophageal squamous cell carcinoma who undergo surgery. The PCR rate with nCRT is higher than that with nCT alone; however, no significant difference in long-term survival has been found between the two approaches (18). This finding suggests that PCR after neoadjuvant therapy does not guarantee a cure for the tumor. Patients who achieved PCR after nCRT were followed up for 3 years after surgery. Among the 47 patients in the nCRT group, eight experienced tumor recurrence, and six died. Although a good PCR rate was observed, no significant improvement in long-term survival rates was noted. Regarding nICT, a propensity score study conducted by Zhao et al. (23) revealed a higher 1-year DFS rate in the nCRT group than in the nICT group (94.3% vs. 81.8%), and a similar finding was observed for 2-year DFS rates. The KEYSTONE-002 study is a phase III clinical trial aimed at investigating the effectiveness of neoadjuvant immunotherapy and nCRT in treating patients with locally advanced esophageal squamous cell carcinoma (24). This trial can provide insights into the optimal combination strategy for neoadjuvant immunotherapy. The 3-year follow-up results of this study indicate that patients who achieved PCR after nCRT were more likely to experience tumor recurrence. While the 3-year survival rate was not statistically significantly different between the two patient groups, the nICT group exhibited superior outcomes in terms of non-recurrence survival rate, particularly distant recurrence rate. The observed difference may stem from the fact that nCRT, while effective in controlling local tumors, may not prevent the dissemination of cancer cells as effectively as nICT. This consideration acknowledges that immunotherapy can induce durable immune memory, which may enhance patients’ ability to resist tumor recurrence in the long term. However, further research is necessary to confirm these findings.

The present study is limited in that it is retrospective in nature, exhibited selectivity bias, had a short follow-up duration, and had a small sample size. Subsequent investigations should be aimed at validating these findings through prospective, multi-center clinical studies involving larger sample sizes.

In conclusion, our two-center retrospective study suggests that clinicians should consider individual patient characteristics when choosing neoadjuvant therapy options, including age, comorbidities, and preferences, to optimize treatment outcomes. This study highlights that nCRT may achieve a higher PCR rate, though is more likely to cause hematotoxic reactions, while nICT may increase the risk of postoperative pulmonary infection. Clinicians should weigh these factors when making treatment decisions. The survival rates of patients who achieved PCR were similar in both groups. However, nICT may provide additional long-term benefits in terms of recurrence-free survival. Further studies are required to determine whether this translates into improved survival rates and to identify more effective treatment strategies for better patient outcomes.

## Data Availability

The raw data supporting the conclusions of this article will be made available by the authors, without undue reservation.
